# Case Report: *Shewanella Algae* Pneumonia and Bacteremia in an Elderly Male Living at a Long-Term Care Facility

**DOI:** 10.4269/ajtmh.21-0614

**Published:** 2021-11-15

**Authors:** Thomas J. Weiss, Javier J. Barranco-Trabi, Aaron Brown, Tiffany T. Oommen, Victoria Mank, Cameron Ryan

**Affiliations:** ^1^ Uniformed Services University of the Health Sciences, Bethesda, Maryland;; ^2^ Department of Internal Medicine, Tripler Army Medical Center, Honolulu, Hawaii

## Abstract

*Shewanella algae* is a gram-negative, nonfermenting, oxidase-positive, motile bacillus that is ubiquitous in aquatic ecosystems. Human infections are rare and the immunocompromised are left most vulnerable. Risk factors for this infection include exposure to seawater, consumption of raw seafood, and underlying comorbid conditions such as hepatobiliary disease and chronic cutaneous ulcers. Previously documented cases of *S. algae* have involved near drownings, contaminated raw shellfish, or wound exposure to seawater, mud, sand, and sewage. This case study is unique in that it describes *Shewanella* bacteremia without any of these typical preceding exposures. We present a case of *S. algae* pneumonia and bacteremia in an elderly male patient living at a long-term care facility without any recent open water exposure.

## INTRODUCTION

**Figure 1.**
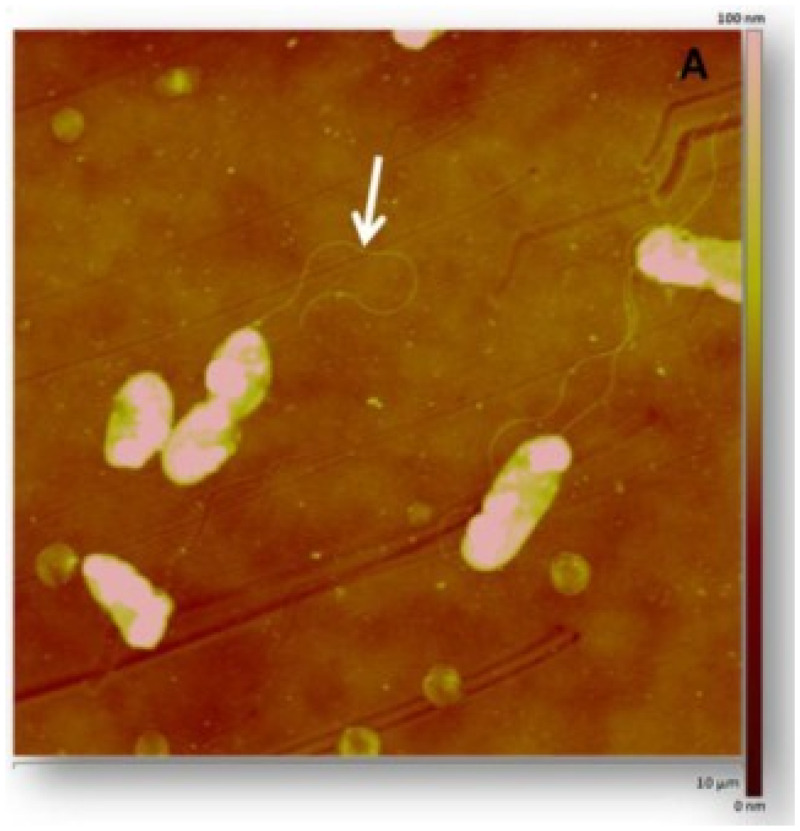
*Shewanella algae* morphology and identifiable singular flagella.^5^ This figure appears in color at www.ajtmh.org.

*Shewanella algae* are heterotrophic facultative anaerobes that are widely distributed in aquatic habitats (Figure 1). Typically, this infection is seen among the immunocompromised population in the setting of open water trauma.^1^^,^^2^ Cases of *Shewanella* have been reported worldwide but are more common in the tropics and during summer months in temperate zones where people are more often in contact with the water.^3^ Previously reported cases suggest that *S. algae* can cause otitis externa, soft tissue infections, bacteremia, and hepatobiliary infections. The organism is often susceptible to third generation cephalosporins, β-lactam/β-lactamase inhibitors, aminoglycosides, and fluroquinolones.^4^ Specific comorbidities associated with *Shewanella* infections include chronic liver and kidney disease, severe peripheral vascular disease (PVD), and skin and soft tissue infections (SSTI).^1^

## CASE REPORT

**Figure 2.**
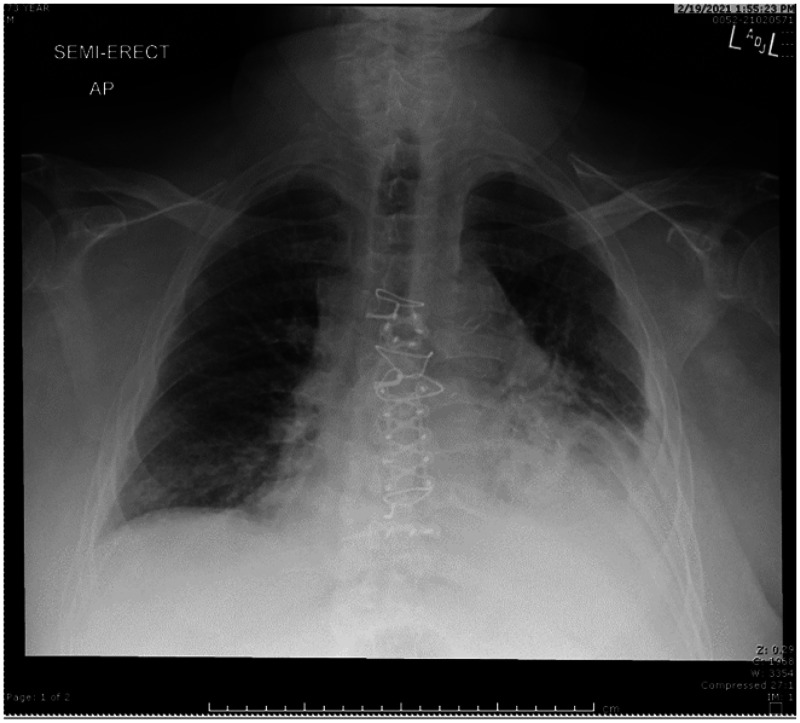
Chest X-ray obtained upon initial presentation. Notable for left lower lobar consolidation with associated pleural effusion, and atelectasis.

Patient is a 73-year-old Caucasian male, with past medical history significant for asthma, chronic obstructive pulmonary disease (COPD), allergic bronchopulmonary aspergillosis, coronary artery disease (CAD), diabetes mellitus type II, and recent acute cholecystitis complicated by sepsis, and treated with a percutaneous cholecystostomy drain. He is a resident of a long-term care facility who initially presented to the emergency department for acute onset of altered mental status, fever (101.4°F), tachycardia (110 bpm), dyspnea, and hypoxia. Initial laboratory evaluation was notable for leukocytosis (19.4) with neutrophil predominance (89.2%), elevated blood urea nitrogen (31), elevated glucose (228), elevated procalcitonin (0.51), and negative COVID-19 test. Chest X-ray demonstrated a left lower lobe pneumonia with associated pleural effusion and atelectasis (Figure 2). Patient was admitted into the hospital with suspected pneumosepsis and empiric antibiotic therapy was initiated with doxycycline and cefepime (to provide broad coverage for methicillin-resistant *Staphylococcus aureus*, *Pseudomonas* and atypical organisms). Blood samples were drawn from the patient and both aerobic cultures resulted in the growth of *S. algae*. Antibiotic susceptibility was assessed via Kirby Bauer disc diffusion assay. The data is displayed in Table 1. The patient improved clinically and the antibiotic regimen was then narrowed to ceftriaxone based on both infectious disease literature and laboratory antibiotic susceptibilities. After completion of the course of antimicrobials, the patient had resolution of all symptoms and was discharged from the hospital.

**Table 1:** Antibiotic sensitivity assessed via Kirby Bauer disc assay*

Antibiotic used	Zone of inhibition (mm)	Sensitivity
Ceftazidime	29	Sensitive
Ciprofloxacin	27	Sensitive
Gentamicin	23	Sensitive
Piperacillin/Tazobactam	30	Sensitive
Tobramycin	21	Sensitive
Imipenem	18	Sensitive
Amikacin	20	Sensitive
Ampicillin	20	Sensitive
Ampicillin/Sulbactam	24	Sensitive
Sulfamethoxazole/Trimethoprim	24	Sensitive
Cefazolin	0	Resistant
Ceftriaxone	26	Sensitive

* MIC results were unable to be obtained.

## DISCUSSION

*Shewanella algae* infections are most common following traumatic injuries and near drownings in both freshwater and saltwater.^6^ Tourists, swimmers, fisherman, and sailors with underlying liver disease and immunocompromising conditions are at highest risk of contracting a water-related soft tissue or pulmonary *Shewanella* infection.^7^^–^^9^ This patient’s exposure to *S. algae* is not easily identified. One potential avenue of infection would be aspiration of a contaminated water supply at the long-term care facility. However, there are no previous case studies describing incidents of aspiration pneumonia caused by *S. algae*. Another possible source of infection would be ingestion of raw seafood, though the patient was unable to recall recent ingestion of any such foods.^10^ Exposure of cutaneous ulcers to seawater is also a well-documented source of infection; however, this patient had no open skin ulcers or seawater exposure in the last 6 months. As such, clinicians should broaden their awareness of this organism’s potential routes of inoculation, predisposing factors for infection, and the wide variety of clinical presentations associated with *S. algae*.

## CONCLUSION

This case presentation describes a unique situation in which a patient develops *S. algae* bacteremia without any identifiable preceding exposures. Though human infection remains rare, the incidence is increasing, and this case highlights the importance of considering *Shewanella* spp. as the cause of infection in settings where the patient may have been exposed to seawater or raw shellfish. This case also brings to light the question of whether potable water may serve as a source of infection and whether additional testing for contamination is indicated.
